# RGD Modification of Poly(2‐oxazoline) Cryogels: Investigation of Material Properties and Cellular Adhesion

**DOI:** 10.1002/mabi.202500421

**Published:** 2026-01-14

**Authors:** Tim Hoffmann, David Pretzel, Steffi Stumpf, Florian Behrendt, Michael Klein, Leon Lange, Lena‐Marie Kaspar, Klaus Liefeith, Michael Gottschaldt, Ulrich S. Schubert

**Affiliations:** ^1^ Laboratory of Organic Chemistry and Macromolecular Chemistry (IOMC) Friedrich Schiller University Jena Jena Germany; ^2^ Jena Center for Soft Matter (JCSM) Friedrich Schiller University Jena Jena Germany; ^3^ Cluster of Excellence Balance of the Microverse Friedrich Schiller University Jena Jena Germany; ^4^ Institute for Bioprocessing and Analytical Measurement Techniques e.V. Heilbad Heiligenstadt Germany

**Keywords:** cell culture, cryogels, poly(2‐oxazoline)s, RGD functionalization, surface modification

## Abstract

In this study, we demonstrate the preparation of a poly(2‐oxazoline) based RGD‐functionalized cryogels and cell culture studies on these cryogels. The present work also involves the investigation of cryogels with ethyl side chains (**CG(B‐Et‐PipA)**) and primary amino groups in their side chain (**CG(B‐Am‐PipA)**) which have been synthesized previously. **CG(B‐Am‐PipA)** was subsequently functionalized with a peptide containing an RGD motif (GCWGRGDSP), resulting in the formation of **CG(B‐Am‐PipA/RGD)**. The coupling was verified by confocal laser‐scanning microscopy (CLSM), fourier‐transform infrared spectroscopy (FT‐IR), high‐resolution magic‐angle‐spinning (HR‐MAS) NMR spectroscopy, and gravimetrical measurements. The degree of functionalization was found to be around 45%. The functionalized gels exhibited a change in their thermal properties, which was examined using thermogravimetric analysis (TGA). Rheological analysis was employed to study the mechanical properties and showed the formation of a stiffer material after peptide coupling. Swelling tests revealed a reduced swelling behavior for **CG(B‐Am‐PipA/RGD)**. Cell biological investigations were conducted with L929 cells which were incubated with the samples **CG(B‐Et‐PipA)RhoB**, **CG(B‐Am‐PipA)RhoB** and **CG(B‐Am‐PipA/RGD)RhoB**. CLSM measurements after 0.5, 1, 2 and 6 h revealed an initial adhesion for both **CG(B‐Am‐PipA)RhoB** and **CG(B‐Am‐PipA/RGD)RhoB**, with the RGD‐functionalized cryogel exhibiting the fastest cell adhesion and the most pronounced adherent phenotype, characterized by distinctly spread cell bodies as confirmed by cell area measurements. Cultivation of cells over a period of 7 days, analyzed by scanning electron microscopy (SEM), further showed pronounced cellular adhesion exclusively on **CG(B‐Am‐PipA)** and **CG(B‐Am‐PipA/RGD)**.

## Graphical abstract

Poly(2‐oxazoline) based cryogels with amino side chains and piperazine derived end groups (**CG(B‐Am‐PipA)**) which were functionalized with an RGD peptide (**CG(B‐Am‐PipA/RGD)**) demonstrated changes in their thermal, mechanical, and swelling properties upon functionalization. Cultivation of L929 cells on the cryogels showed that both **CG(B‐Am‐PipA)** and **CG(B‐Am‐PipA/RGD)** facilitate cellular adhesion and spreading, a phenomenon which was not observed for the pure poly(2‐ethyl‐2‐oxazoline) based gel (**CG(B‐Et‐PipA)**).

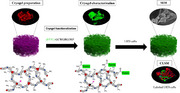

## 1 Introduction

Cryogels are sponge‐like materials with an interconnected macroporous structure that ensures mechanical integrity while supporting effective fluid flow and molecular diffusion. In combination with a precisely tailorable surface functionalization these materials are very promising in the field of tissue engineering due to their capability to promote the adhesion and the spreading of cells by mimicking the open‐pored architecture of the natural extra‐cellular matrix (ECM) and allowing for the supply with nutrients [1]. Cryogels designed as ECM mimics are typically comprised of natural biopolymers such as gelatin [2, 3], cellulose or chitosan [4, 5], due to their excellent biocompatibilities, which are known to demonstrate cell‐adherent behavior and cell‐spreading [6].

On the other hand, synthetic polymers such as poly(ethylene glycol) diacrylate (PEGDA) or poly(vinyl alcohol) (PVA) offer increased mechanical stability. Thus, cryogels are often also prepared based on a combination of both synthetic and natural polymers, allowing for a high biocompatibility and mechanical stability [7, 8, 9]. In particular, cryogels containing bioactive molecules have gained a lot of interest within the last years which can either be introduced directly into the polymeric network using functional monomers or cross‐linker(s) [10], or *via* post‐fabrication modification reactions by the exploitation of certain functional groups on the cryogel surfaces [11, 12]. The review of Behrendt et al. provides an extensive overview about different types of functionalization strategies for the development of functional cryogels as well as analytical methods for proving the successful functionalization [13].

Poly(2‐oxazoline)s (POx) represent a class of synthetic polymers which are characterized by their biocompatibility, stealth properties, protein‐like structure, and synthetic structural diversity [14, 15]. For instance, by the use of an appropriate bifunctional initiator and polymerizable end capping agents, POx based cross‐linkers can be obtained which can also be used for cryogel preparation [16, 17]. Functional oxazolines such a [3‐[(*tert*‐butoxycarbonyl)amino]propyl]‐4,5‐dihydrooxazole (BocOx) or methyl 3‐(4,5‐dihydrooxazol‐2‐yl)propanoate (MestOx) direcly enable the introduction of reactive groups in the side chains of the polymeric network which can be further modified after the cryogel fabrication [18].

Based on our previous results about the preparation of cryogels containing 2‐ethyl‐2‐oxazoline (EtOx) and amino‐functionalized oxazoline (AmOx), we report in this work the preparation and characterization of RGD‐functionalized POx based cryogels. Confocal laser‐scanning microscopy (CLSM), fourier‐transform infrared spectroscopy (FT‐IR), high‐resolution magic‐angle spinning (HR‐MAS) NMR spectroscopy, and gravimetrical measurements were used to prove the successful functionalization. Furthermore, we investigated the changes of the mechanical, thermal, and swelling properties in the course of the functionalization. The adhesion properties of L929 cells on cryogels with different surface functionalizations were investigated using scanning electron microscopy (SEM) and CLSM which revealed adherent cells in the case of the RGD‐functionalized gels.

## 2 Results and Discussion

### 2.1 Cryogel Preparation

**SCHEME 1:**
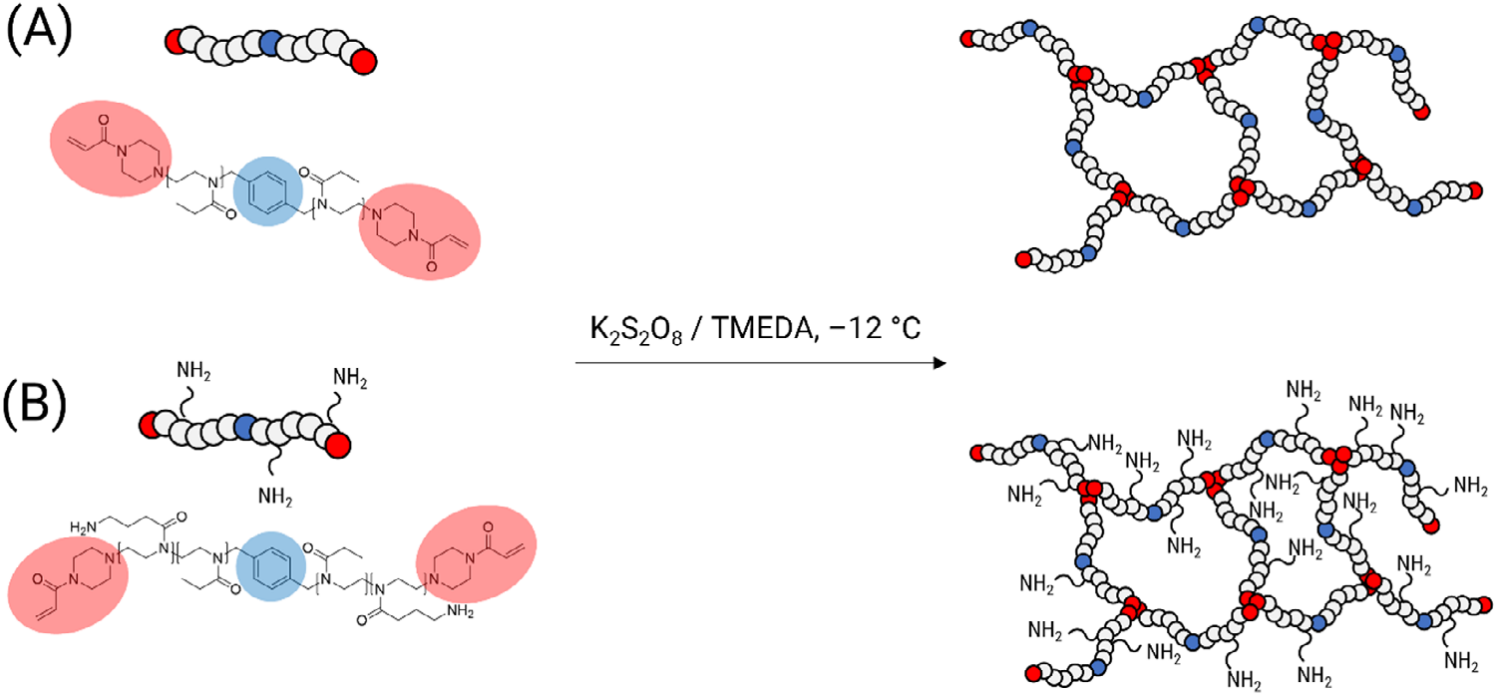
Schematic representation of the cryogel synthesis by cryo‐polymerization at ‒12 °C using a 2‐ethyl‐2‐oxazoline (EtOx)‐based cross‐linker (**B‐Et‐PipA**, (A)) or using an amino propyl functionalized oxazoline (AmOx) based cross‐linker (**B‐Am‐PipA**, (B)).

The cryogel monoliths were prepared according to the literature without the use of acryloxyethyl thiocarbamoyl rhodamine B (RhoB) [16, 18]. For this purpose, the bifunctional (B) poly(2‐oxazoline) (POx)‐based cross‐linkers were utilized, which had been synthesized and characterized previously (Scheme 1) [16]. These cross‐linkers consisted solely on 2‐ethyl‐2‐oxazoline (EtOx) (Et) or in combination with amino propyl functionalized oxazoline (AmOx) units (Am), with polymerizable piperazine‐acrylamide (PipA) ω‐end groups and were named **B‐Et‐PipA** or **B‐Am‐PipA**. The cross‐linkers were utilized in a free radical polymerization at subzero temperatures of ‒12°C, with K_2_S_2_O_8_/*N*,*N*,*N’*,*N’*‐tetramethylethylenediamine (TMEDA) acting as radical initiator pair. The exact experimental details are summarized in Table S1. The resulting cryogels **CG(B‐Et‐PipA)** and **CG(B‐Am‐PipA)** were obtained as stable monoliths. Previously published cryogels containing approximately 0.2 mol% of the fluorescent comonomer RhoB were utilized to exclusively demonstrate the successful functionalization reaction and subsequent visualization of the L929 cells within the porous structure of the gels via confocal laser‐scanning microscopy (CLSM) [18]. These gels were designated as **CG(B‐Et‐PipA)RhoB** and **CG(B‐Am‐PipA)RhoB**.

### 2.2 Cryogel Functionalization

**SCHEME 2:**
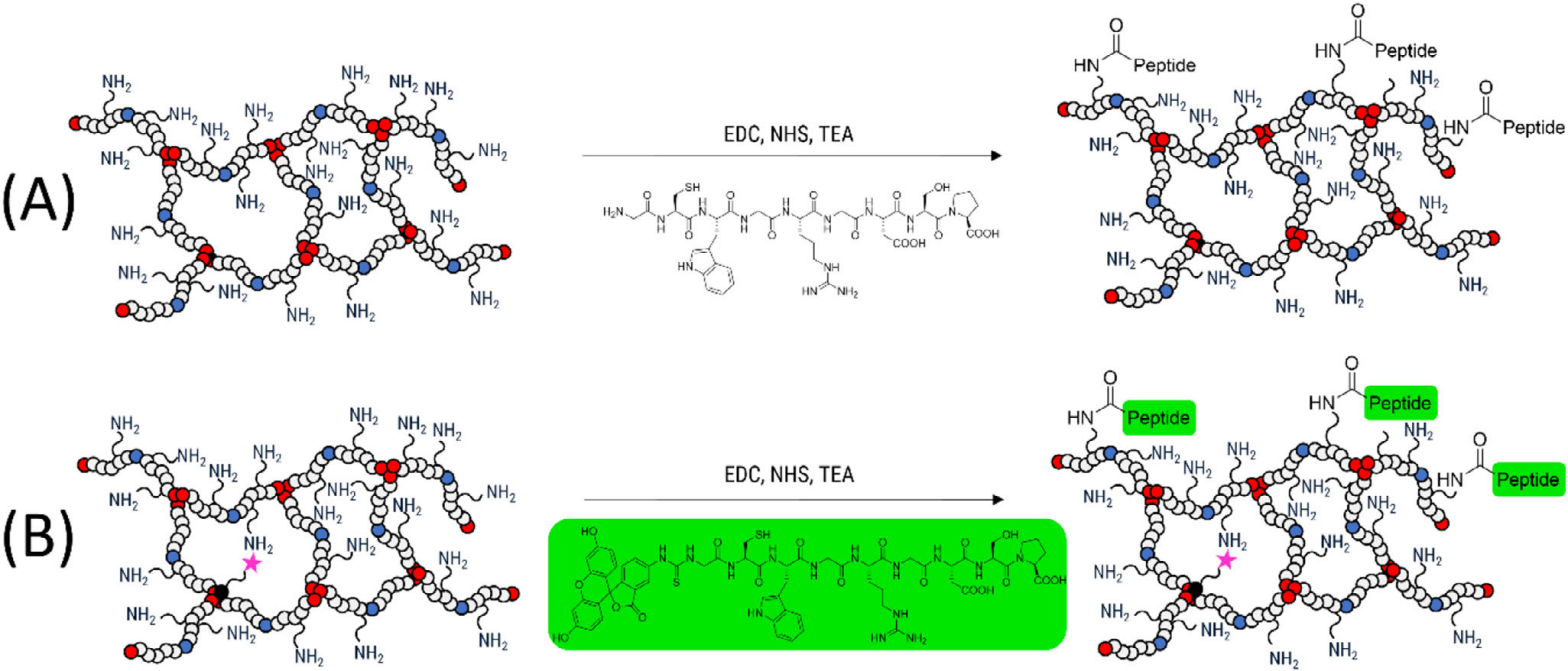
Schematic representation of the cryogel functionalization using **CG(B‐Am‐PipA)** and a RGD containing peptide (GCWGRGDSP) (A). The resulting cryogels are designated as **CG(B‐Am‐PipA/RGD**). To facilitate the assessment of the peptide coupling by fluorescence microscopy, the same peptide sequence containing a fluorescein label ((FITC)‐GCWGRGDSP) was coupled to fluorescent cryogels containing approx. 0.2 mol% of acrylthiocarbamoyl rhodamine B (RhoB) (**CG(B‐Am‐PipA)RhoB**) (B). The resulting cryogels are designated as CG(**B‐Am‐PipA/FITC‐RGD)RhoB**.

The functionalization of **CG(B‐Am‐PipA)** was achieved by using of the following peptide sequence: GCWG**RGD**SP (**RGD**) (Scheme 2A). In a study by Tsurkan et al., the adhesive behavior of hydrogels was demonstrated for this exact peptide sequence [19]. However, this approach has yet to be implemented in the context of cryogels. A reaction solution comprising 1‐ethyl‐3‐(3‐dimethylaminopropyl)carbodiimide hydrochloride (EDC‐HCl), *N*‐hydroxysuccinimide (NHS), and **RGD** was prepared and was partially added to the cryogel until complete swelling was reached. The use of the coupling reagents EDC‐HCl and NHS facilitates the activation of the acid groups of **RGD** by the formation of an NHS ester which reacts with the amino‐containing gel to form covalent amide bonds with the gel, designated as **CG(B‐Am‐PipA/RGD)**. To avoid side reactions, such as the COOH group of the RGD peptide reacting with its own N‐terminus, the reaction solution was added directly to the cryogels. Additionally, the ratio between RGD and free amine groups of the cryogel was set to around 1:3.75 to increase the probability of coupling to the cryogel. Following the coupling process, the gels underwent multiple washing steps with water and triethylamine (TEA) to ensure the removal of unreacted peptide and byproducts. For proof of functionalization via CLSM imaging, a dried sample of **CG(B‐Am‐PipA)RhoB** was functionalized with **RGD** containing a fluorescein isothiocyanate (FITC) moiety at its *N*‐terminus (FITC‐GCWG**RGD**SP, designated as **FITC‐RGD**) resulting in **CG(B‐Am‐PipA/FITC‐RGD)RhoB** (Scheme 2B).

**FIGURE 1:**
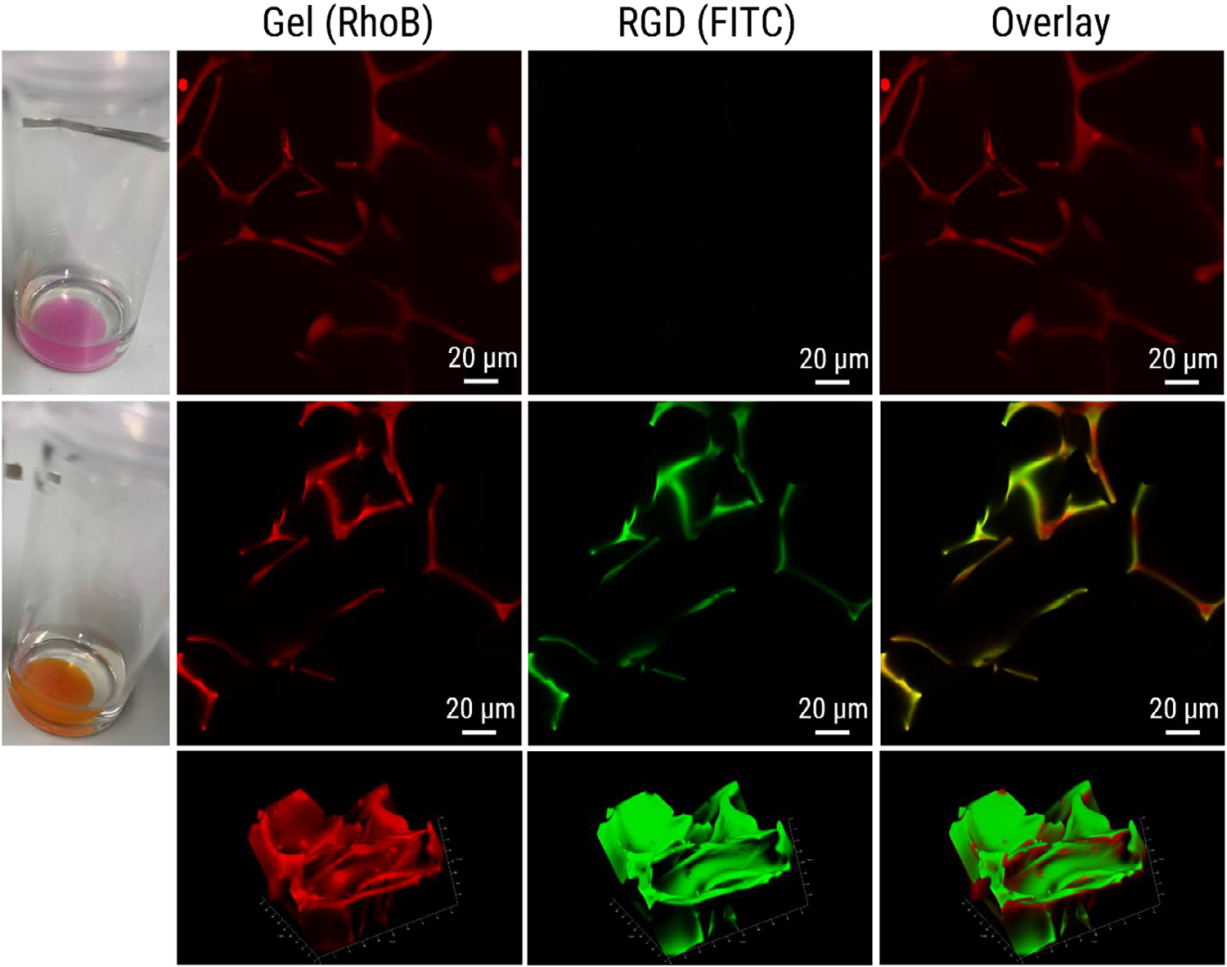
Overview of cryogel pictures (left column) and CLSM images for different channels (middle columns and right column). **CG(B‐Am‐PipA)RhoB** before functionalization with FITC‐RGD (upper row), resulting in **CG(B‐Am‐PipA/FITC‐RGD)RhoB** (middle row). CLSM images represent the RhoB channel (middle left column), the FITC channel (middle right column) and the overlay of both channels (right column). In the bottom row, 3D CLSM images of the cryogel are displayed.

As illustrated in Figure 1, the CLSM images from swollen **CG(B‐Am‐PipA)RhoB** (upper row) and **CG(B‐Am‐PipA/FITC‐RGD)RhoB** (middle row) reveal the porous nature of the cryogels, which can be seen in the rhodamine channel, indicating that the porous structure remained intact even after the coupling process. As expected, no signal in the FITC channel was detected for **CG(B‐Am‐PipA)RhoB** indicating that the RhoB label is not interfering with the signal of FITC when using this particular channel. For **CG(B‐Am‐PipA/FITC‐RGD)RhoB**, a signal in this channel was observed which can be assigned to the FITC fluorophore, indicating a successful coupling of **FITC‐RGD** to the cryogel. As demonstrated by the overlay of the FITC and RGD channels, the colocalized presence of the FITC‐RGD and RhoB signals in cryogel material suggests a successful coupling. Additional software image analysis revealed an overlap with a pearson  coefficient of 0.51 (Figure S1). This is further confirmed by the 3D representation of **CG(B‐Am‐PipA/FITC‐RGD)RhoB**, which provides additional insights into the cryogel's pore structure (Figure 1, bottom row). Furthermore, a visible color change is evident in the cryogels themselves over the course of the functionalization reaction (Figure 1, left column). Prior to the functionalization process, the gel exhibits a pink coloration; however, after the functionalization, it acquires an orange color. Despite undergoing multiple washing cycles, the color remains unchanged, which is indicative of successful coupling.

**FIGURE 2:**
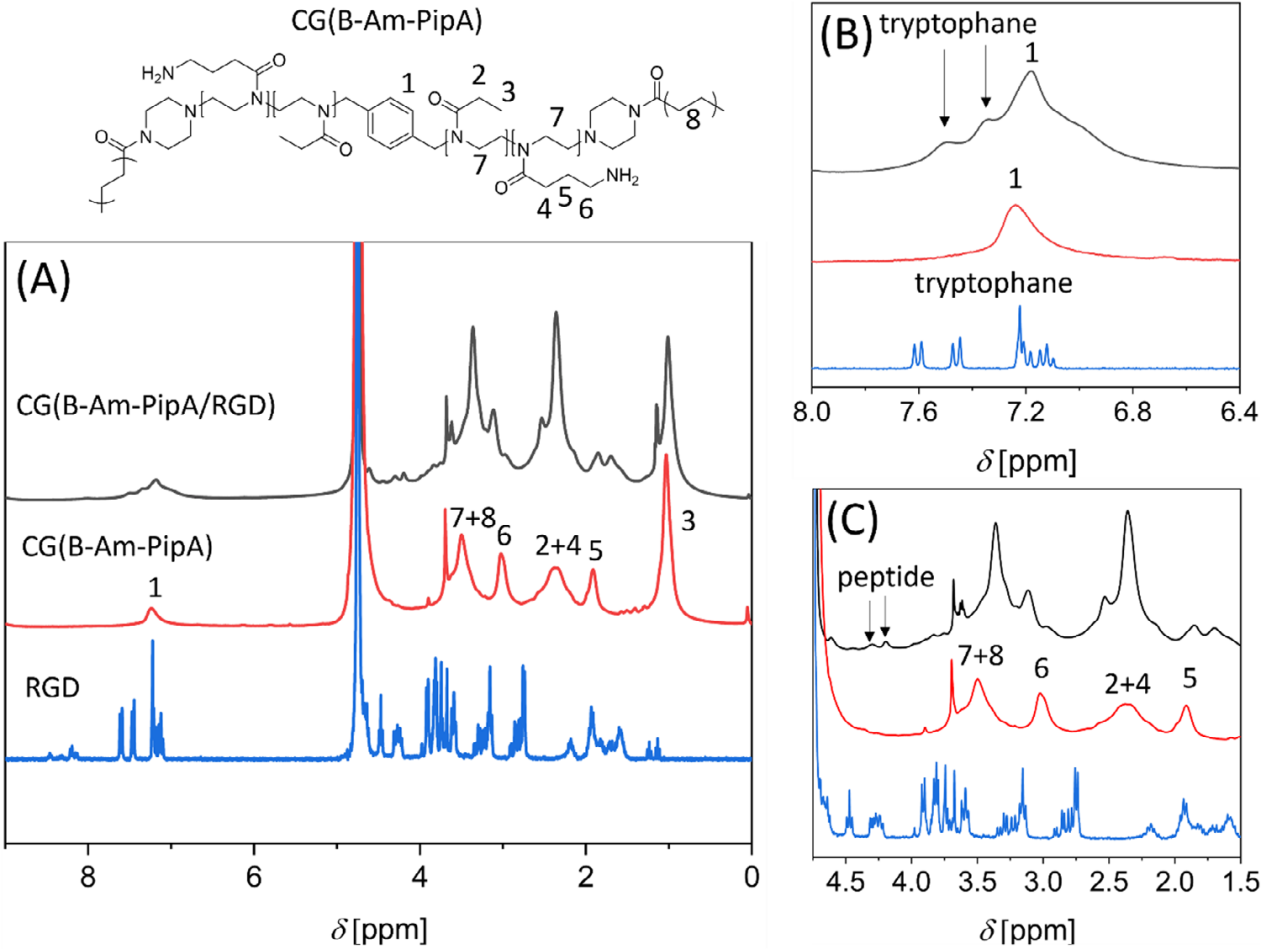
Overlay of HR‐MAS NMR spectra of **CG(B‐Am‐PipA/RGD)** (black), **CG(B‐Am‐PipA)** (red), and GCWGRGDSP (RGD, blue). Full spectra (A) as well as zoom‐ins into the aromatic region (B) or into the polymer and peptide related region (C) are displayed.

For the non‐RhoB labelled cryogels the chemical composition of the cross‐linked structures was analyzed using HR‐MAS NMR (Figure 2A, red curve). In the ^1^H NMR spectrum, **CG(B‐Am‐PipA)** exhibited characteristic peaks for the used cross‐linker **B‐Am‐PipA** at around 1.0 and 2.4 ppm, which can be assigned to the CH_3_ and CH_2_ groups of the EtOx side chains, respectively [16]. Additionally, the three CH_2_ signals of the AmOx side chains at 1.9, 2.4, and 3.0 ppm can be observed. The peak at 3.5 ppm indicates the presence of both the cryogel backbone and the POx backbone. Double‐bond signals, which can be attributed to the initial cross‐linker **B‐Am‐PipA**, are no longer present. This observation indicates that either the non‐reacted cross‐linker was removed during the washing steps or that the conversion rate was approaching 100%. After the coupling reaction, the CH_2_ groups of the AmOx side chain revealed a shift for **CG(B‐Am‐PipA/RGD)**, indicating that the RGD was coupled to the amino groups. Additionally, there are signals in the aromatic region from 6.5 to 7.7 ppm, which are indicative of the tryptophane moiety of the peptide (Figure 2B). The presence of additional peptide signals between 1.5 and 4.5 ppm further corroborates the success of the coupling (Figure 2C). The quantity of coupled RGD was determined gravimetrically by weighing the samples in the dried state prior to the reaction. Following the reaction and several washing steps, the samples were dry weighed again to obtain the amount of **RGD** that had been coupled. Accordingly, the number of **RGD** molecules per POx chain ranged from 0.80 to 0.85 for all coupled gels, corresponding to a mole fraction of 44% to 46% (Table S2). A comparable amount of **RGD** per polymer chain was found for all cryogels, indicating a consistent reaction.

**FIGURE 3:**
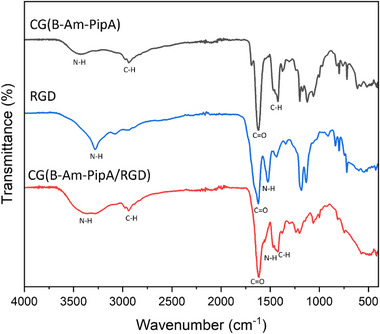
Overlay of FT‐IR spectra of **CG(B‐Am‐PipA)** (red), GCWGRGDSP (RGD, blue), and **CG(B‐Am‐PipA/RGD)** (black), indicating characteristic signals of the amide and amine vibrations at 1624 cm^−1^ (v(C═O)) and 3100 to 3500 cm^−1^ (v(N–H)), respectively.

By comparing the FT‐IR spectra of the initial materials **CG(B‐Am‐PipA)** (black line) and RGD (blue line) as well as the functionalized **CG(B‐Am‐PipA/RGD)** (red line), the success of the RGD functionalization was additionally proven (Figure 3). The presence of a narrow band at 1624 cm^−1^ was detected for **CG(B‐Am‐PipA)** and can be attributed to the carbonyl vibration (v(C═O)) of the amide group of the POx backbone. Furthermore, the signal at 3421 cm^−1^ can be assigned to the primary amino group of the AmOx side chain (v(N–H)). Consequently, it can be concluded that the incorporation of the cross‐linker **B‐Am‐PipA** into the 3D network of the cryogel was successful. The **CG(B‐Am‐PipA/RGD)** exhibited the characteristic v(C═O) band, which can be attributed to the amide groups of RGD and of the gel itself. Additionally, a broader and more intense band at 3100–3500 cm^−1^ is evident in comparison to **CG(B‐Am‐PipA)** (black) and RGD (blue). This observation can be attributed to the NH‐amide vibrations of the peptide itself and the amides that are formed during the coupling process.

### 2.3 Physical Properties of Cryogels

**FIGURE 4:**
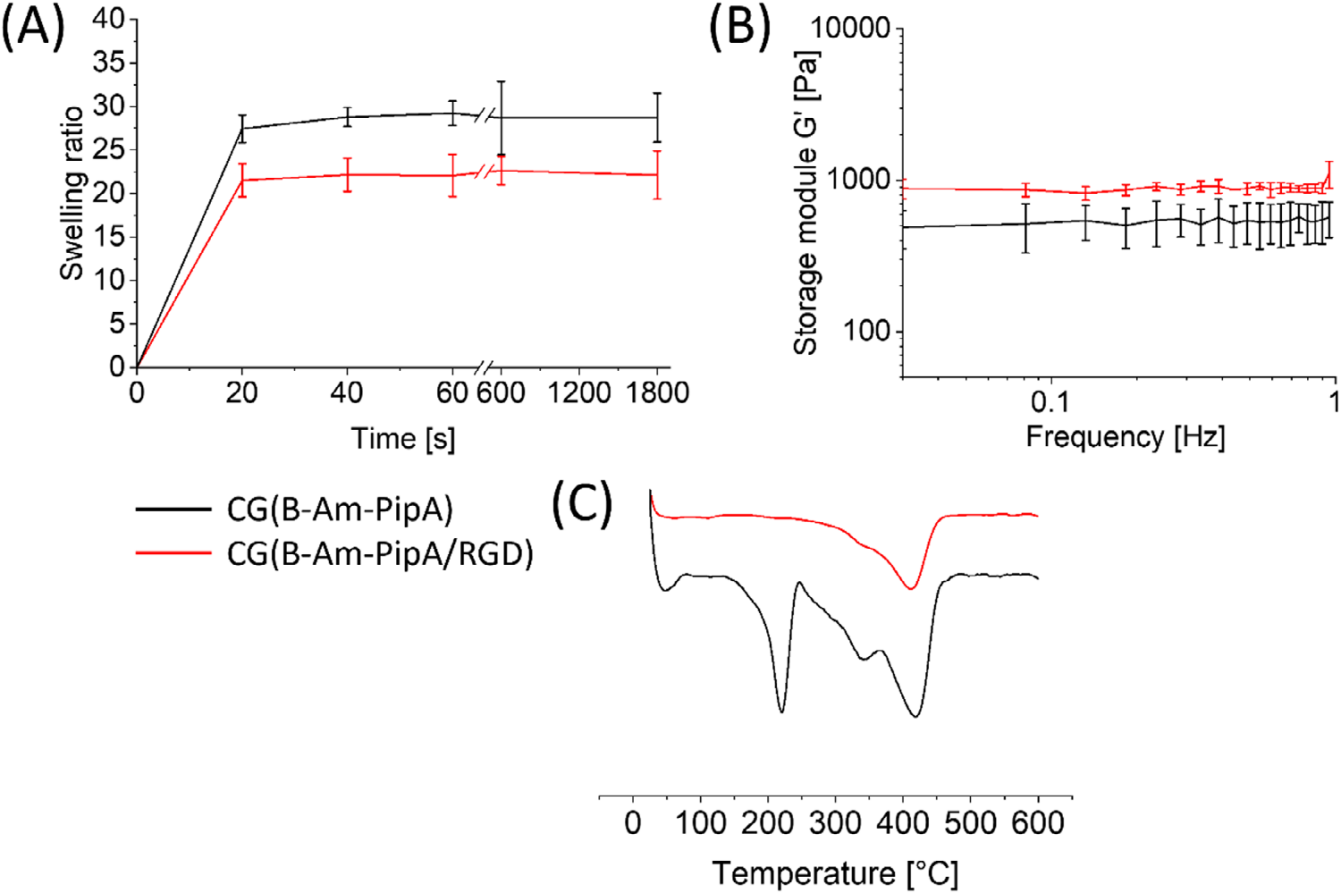
Overview of the results from swelling measurements, rheological measurements, and TGA analyses of **CG(B‐Am‐PipA)** (black curves) and **CG(B‐Am‐PipA/RGD)** (red curves). Swelling experiments for the determination of the swelling ratios were performed in triplicates (A). Frequency sweep measurements were performed at 25 °C in triplicates (B). First derivatives of TGA degradation curves are displayed (C).

As illustrated in Figure 4, a comparison of the physical properties of the cryogels before and after the RGD functionalization has been conducted. To determine the swelling properties of the different cryogels, the dry cryogels were weighed and subsequently swollen in water. After a defined period of time, they were weighed again. The gels' swelling properties (Figure 4A) revealed that the maximum swelling ratio of 29 and 22 is already reached after 20 s for both **CG(B‐Am‐PipA)** and **CG(B‐Am‐PipA/RGD)** (Figure 4A). The reason for the observed reduction in swelling upon RGD functionalization can be attributed to the amides that are formed during the reaction. Consequently, the amount of primary amino units that drive the high swelling capacity of **CG(B‐Am‐PipA)** are reduced. Additionally, the mechanical properties of both materials were investigated through rheological measurements (Figure 4B). Therefore, we used the cone plate setup, which is widely applied in the literature and allows cryogels to be measured without damaging them. Both cryogels were stable within the frequency range of 0.03 to 1.0 Hz. In comparison, **CG(B‐Am‐PipA/RGD)** (920 Pa) demonstrated a 1.6 higher storage modulus than **CG(B‐Am‐PipA)** (570 Pa). We presume that this might be due to the additional cross‐linking within the cryogel structure. Given the presence of two free acid groups within the RGD peptide sequence, the peptide itself exhibits the capacity to react twice with the gel via both groups. This cross‐linking reaction contributes to the formation of a stiffer material. It is known from the literature that additional cross‐linking subsequently leads to stiffer gels [20]. Due to its comparable mechanical properties, **CG(B‐Am‐PipA/RGD)** could be used e.g. as artificial lung tissue in tissue engineering [21]. In order to investigate the thermal properties and potential stability of the gels during autoclaving, thermogravimetric analysis (TGA) was performed (Figure 4C). An initial thermal transition can be observed for **CG(B‐Am‐PipA)** at approximately 220 °C, which indicates the degradation of the trifluoroacetate (TFA) anion [22]. However, in the case of **CG(B‐Am‐PipA/RGD)**, this transition is no longer evident which can be explained by the complete removal of the TFA salt from the gel and the peptide due to deprotonation of the ammonium groups by excessive washing steps with TEA. For **CG(B‐Am‐PipA)**, a second transition at approximately 340 °C was observed which decreased after the peptide coupling. Consequently, it can be concluded that this might be caused by the decomposition of the amino groups. As the amount of free amino groups in the cryogel is reduced upon the RGD functionalization, a high conversion of the functionalization will consequently lead to a reduction of the transition signal intensity. The remanence of a small transition indicates that not all amino groups were functionalized. At 420 °C, a significant transition is evident, related to the decomposition of the cryogel backbone and the peptide groups [23, 24].

### 2.4 Cell Culture Studies

**FIGURE 5:**
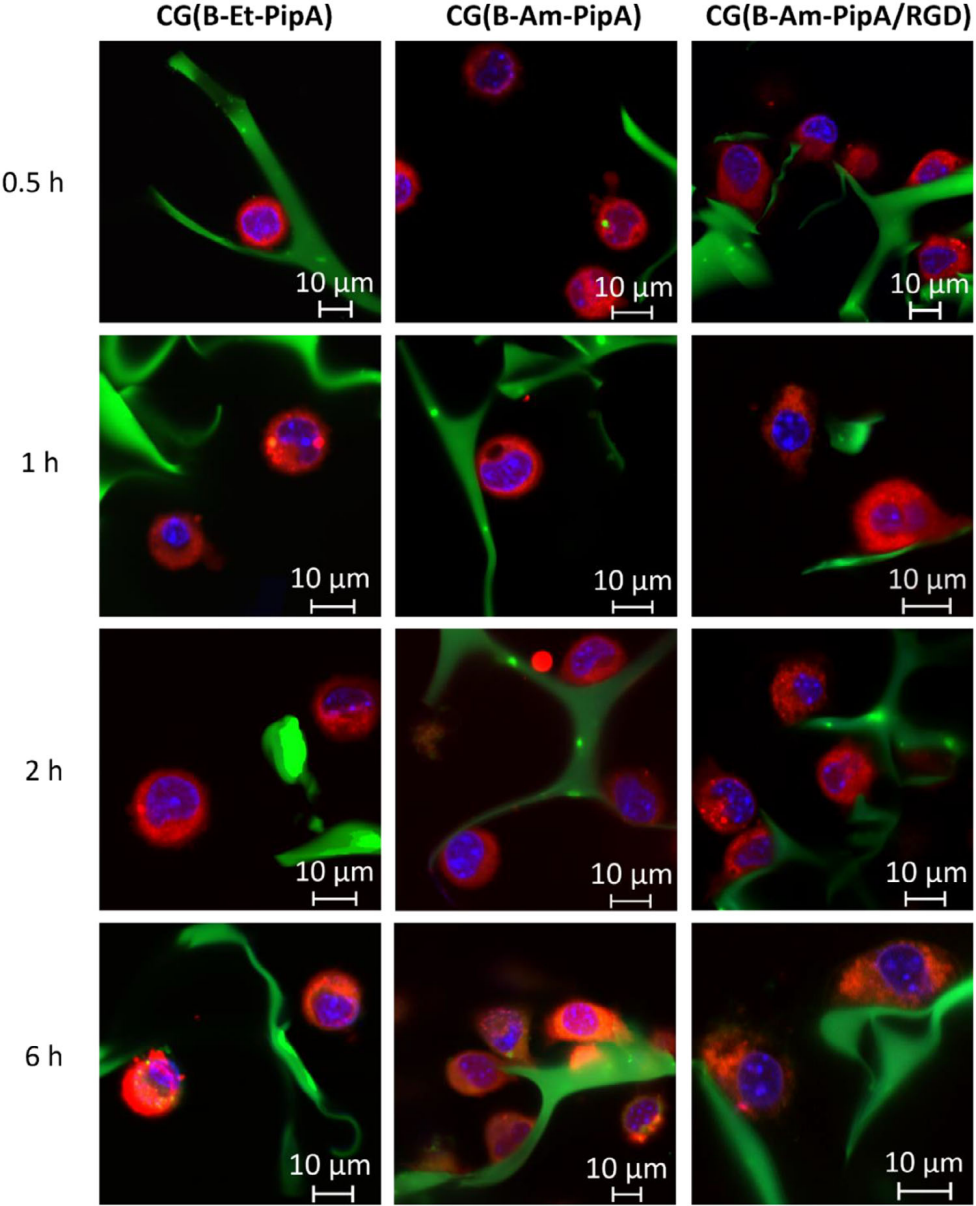
CLSM images of L929 cell adhesion on **CG(B‐Et‐PipA)RhoB**, **CG(B‐Am‐PipA)RhoB,** and **CG(B‐Am‐PipA/RGD)RhoB** after 0.5, 1, 2, and 6 h of incubation. Cell plasma was labelled using CTDR (red), the cell nucleus was stained with Hoechst 33258 (blue) and cryogels were conjugated with RhoB (green).

To investigate the adhesion of L929 cells on different functionalized cryogel surfaces, **CG(B‐Et‐PipA)RhoB**, **CG(B‐Am‐PipA)RhoB**, and **CG(B‐Am‐PipA/RGD) RhoB**, were selected for comparison. To investigate the initial cell‐response, CLSM measurements were performed 0.5, 1, 2, and 6 h after initial cell seeding on top of the cryogels (Figure 5). For **CG(B‐Et‐PipA)RhoB**, cells with round morphologies and no apparent adhesion on the cryogel matrix were observed at all time points, indicating no cell‐cryogel interaction. In the case of **CG(B‐Am‐PipA)RhoB**, cells also exhibited round morphologies after 0.5 and 1 h. However, after 2 h, cells began to interact with the cryogel and appeared to attach. After 6 h, clear attachment and cell flattening were observed, suggesting progressive adhesion over time. The presence of a cationic surface with respect to amino units could be a decisive factor in this phase of cell attachment, resulting in the formation of numerous adhesion structures. The electrostatic charge of the surface might play an important role in the initial settling stage, wherein the negative charge of the cell membrane interacts with the cationic surface of the gel [25, 26]. In contrast to this **CG(B‐Am‐PipA/RGD)RhoB** sample displayed flat shaped cells on the cryogel surface already after 0.5 h, indicating the fastest adhesion among the tested cryogels. This demonstrates that the presence of **RGD** promotes and enhances the initial cell adhesion process.

**FIGURE 6:**
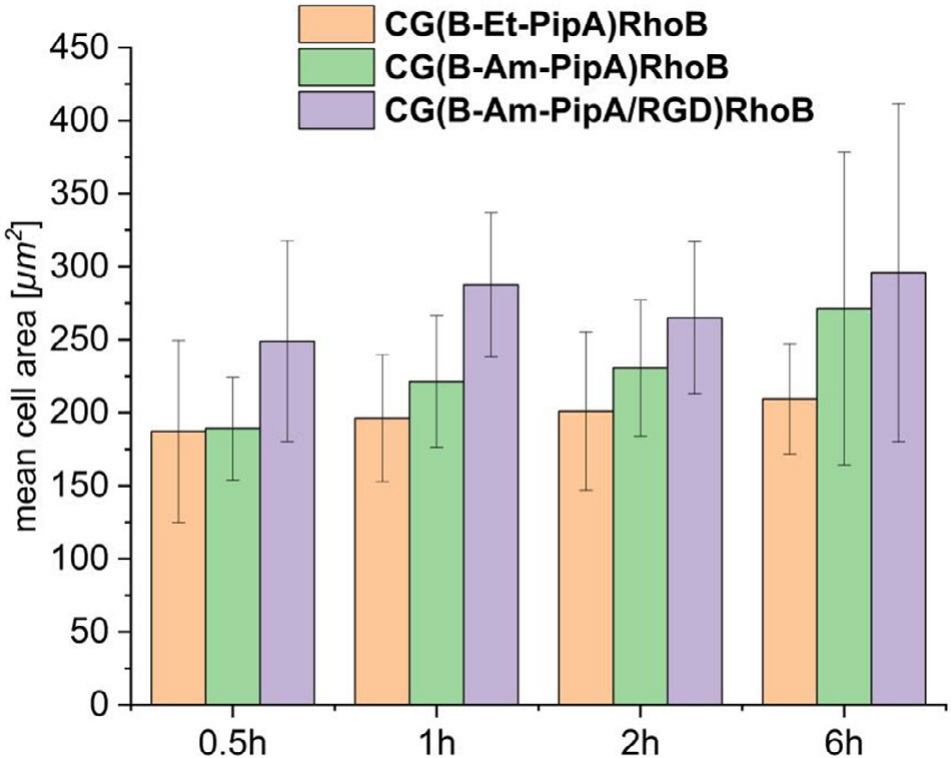
Mean cell area of cells cultured on **CG(B‐Et‐PipA)RhoB**, **CG(B‐Am‐PipA)RhoB**, and **CG(B‐Am‐PipA/RGD)RhoB** cryogels after 0.5, 1, 2, and 6 h.

These findings could be confirmed by image analysis estimating the cell area, revealing the biggest cell area for all time points for **CG(B‐Am‐PipA/RGD)RhoB** followed by **CG(B‐Am‐PipA)RhoB** and **CG(B‐Et‐PipA)RhoB** (Figure 6). The cell area for **CG(B‐Et‐PipA)RhoB**, kept constant over all time points, which is consistent with the microscopy images, where the cells maintained a round morphology and showed no adhesion to the cryogel. The **CG(B‐Am‐PipA)RhoB** sample exhibited similar cell area values as **CG(B‐Et‐PipA)RhoB** after 0.5 h. However, the cell area increases with time, indicating cell flattening and hence an adhesion of the to the cryogel. **CG(B‐Am‐PipA/RGD)RhoB** already shows higher cell area values after 0.5 h compared to the other samples, indicating that cell flattening occurs the fastest on this cryogel.

**FIGURE 7:**
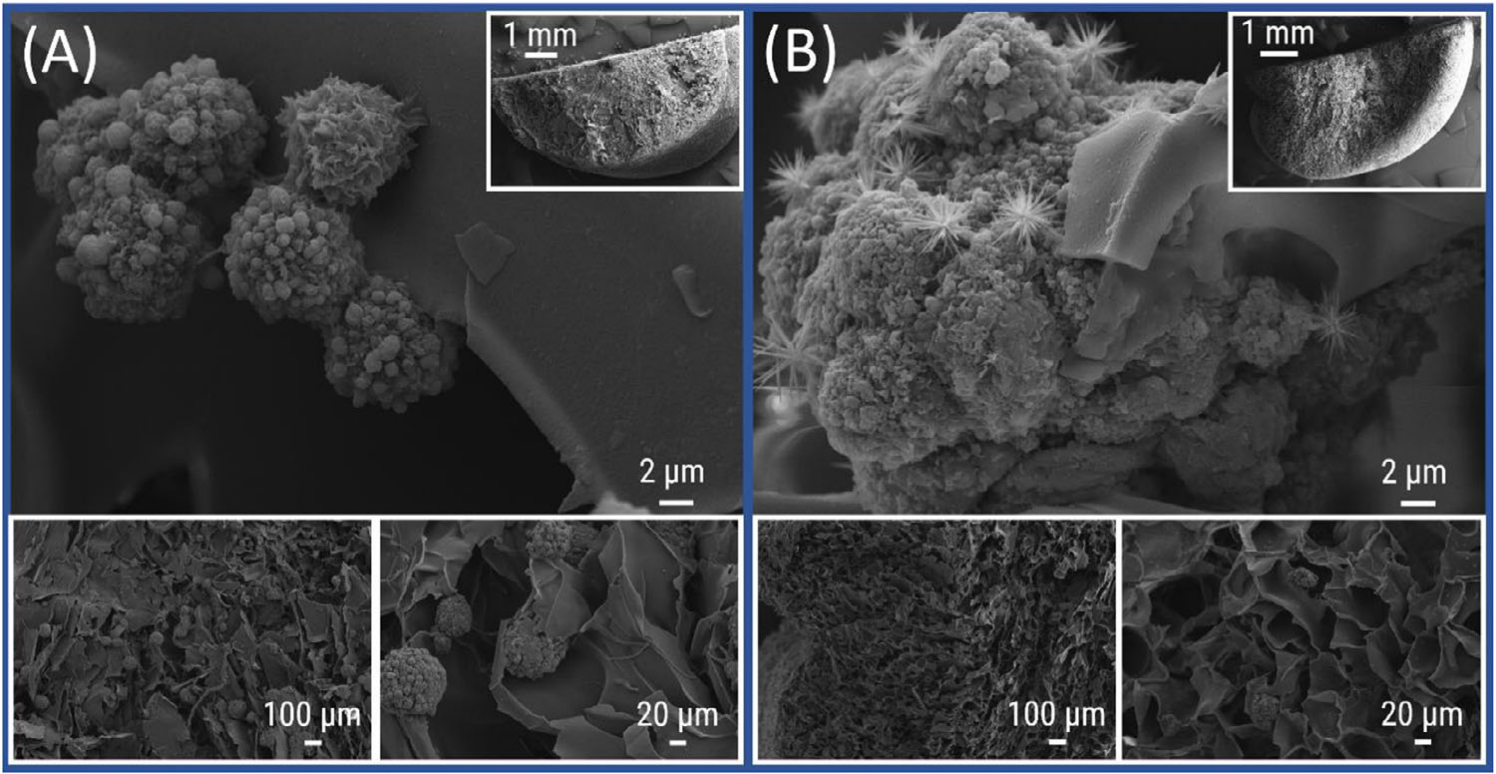
SEM micrographs of **CG(B‐Et‐PipA)** with different magnifications containing L929 cells which were fixed directly after cell culture on unwashed gels (A) or gels which were washed after the cell culture prior to the fixation (B) (A. top: 2500×; top right: 14×; bottom left: 50×; bottom right: 250×), (B. top: 2500×; top right: 16×; bottom left: 50×; bottom right: 250×).

To investigate the behavior of the cells after a longer period a detailed examination of the cell morphology after seven days of culture with the cryogels was performed by SEM analysis. The cells were fixed either directly with glutaraldehyde or after washing with a phosphate‐buffered saline (PBS) solution in order to investigate if the cells firmly adhered to the gel surface. After fixation, the gels were immersed in a PBS buffer solution following an ethanol series (30%, 50%, 70%, 90%, 100%) for dehydration and finally dried using a critical point dryer (CPD). Following this procedure, the gels were cut in half along its central axis and the top and bottom sections were examined separately by SEM. Cells cultured on **CG(B‐Et‐PipA)** only rarely occur on the unwashed gel and show round morphologies with weakly pronounced focal adhesions (Figure 7A). This low cell density is further decreased by the additional washing step as displayed in Figure 7B, suggesting that there was no strong interaction between adhesion‐mediating cell components and the hydrophilic polymer network of the cryogel material. A few cell spheroids are found demonstrating cell‐to‐cell, but not cell‐to‐cryogel interactions. The image in Figure 7B additionally depicts the presence of crystalline structures, which could be attributed to components of the cell cultivation medium, since these structures have also been observed in gels cultured with cell‐free medium (Figure S2).

**FIGURE 8:**
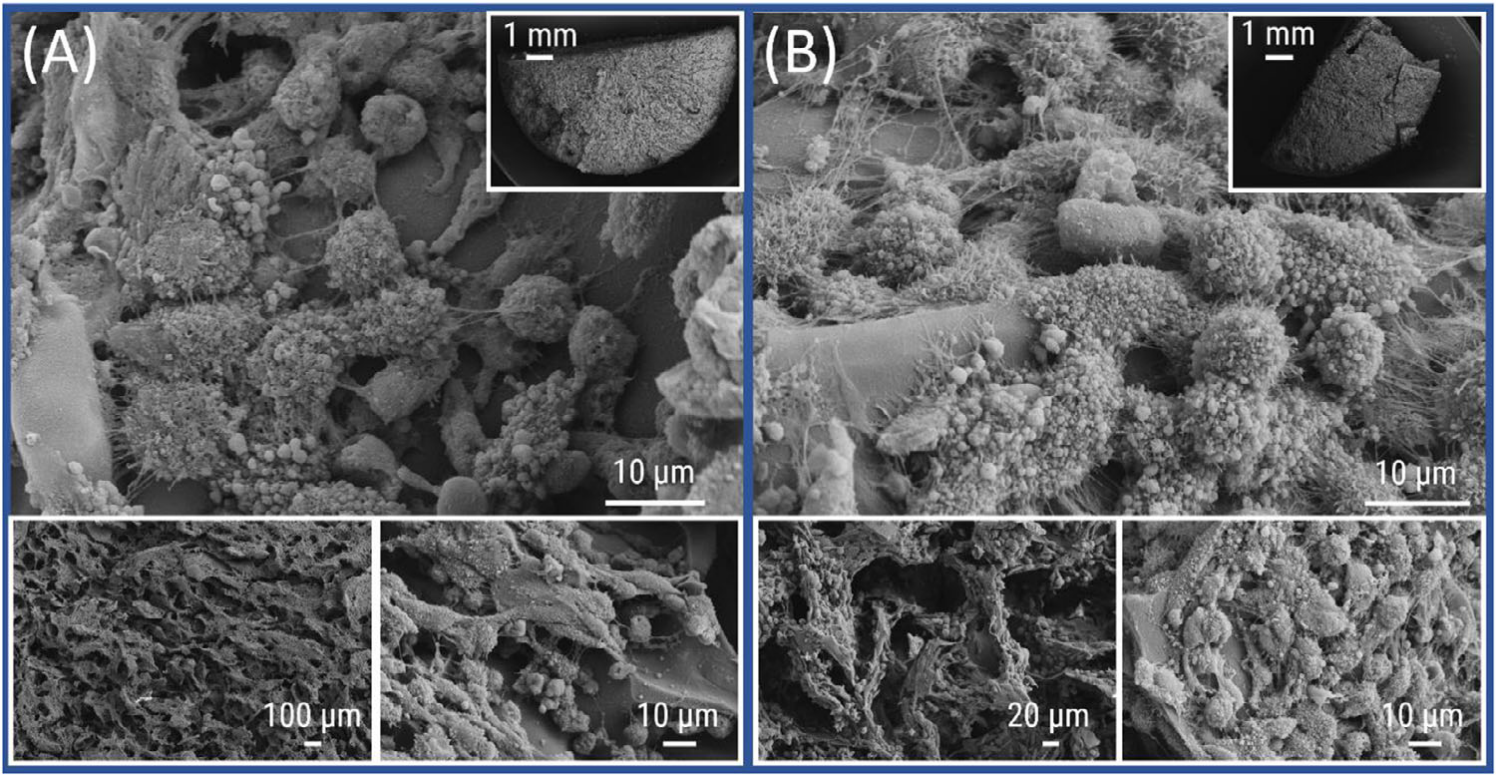
SEM micrographs of **CG(B‐Am‐PipA)** with different magnifications containing L929 cells which were fixed directly after cell culture on unwashed gels (A) or gels which were washed after the cell culture prior to the fixation (B) (A. top: 1500×; top right: 12×; bottom left: 50×; bottom right: 1000×), (B. top: 1600×; top right: 12×; bottom left: 250×; bottom right: 1000×).

In contrast to **CG(B‐Et‐PipA)**, cell culture studies on **CG(B‐Am‐PipA)** clearly revealed cellular attachments in both the unwashed (Figure 8A) and the washed (Figure 8B) sample indicating a relatively strong formation of adhesion points between cells and the cryogel. While the cells were evenly distributed across the gel surface, their morphology reveals the presence of numerous well‐developed filopodia. Additionally, the typical flat‐shaped phenotype of adherent cells and the round shape of cells before or after cell division can be observed.

**FIGURE 9:**
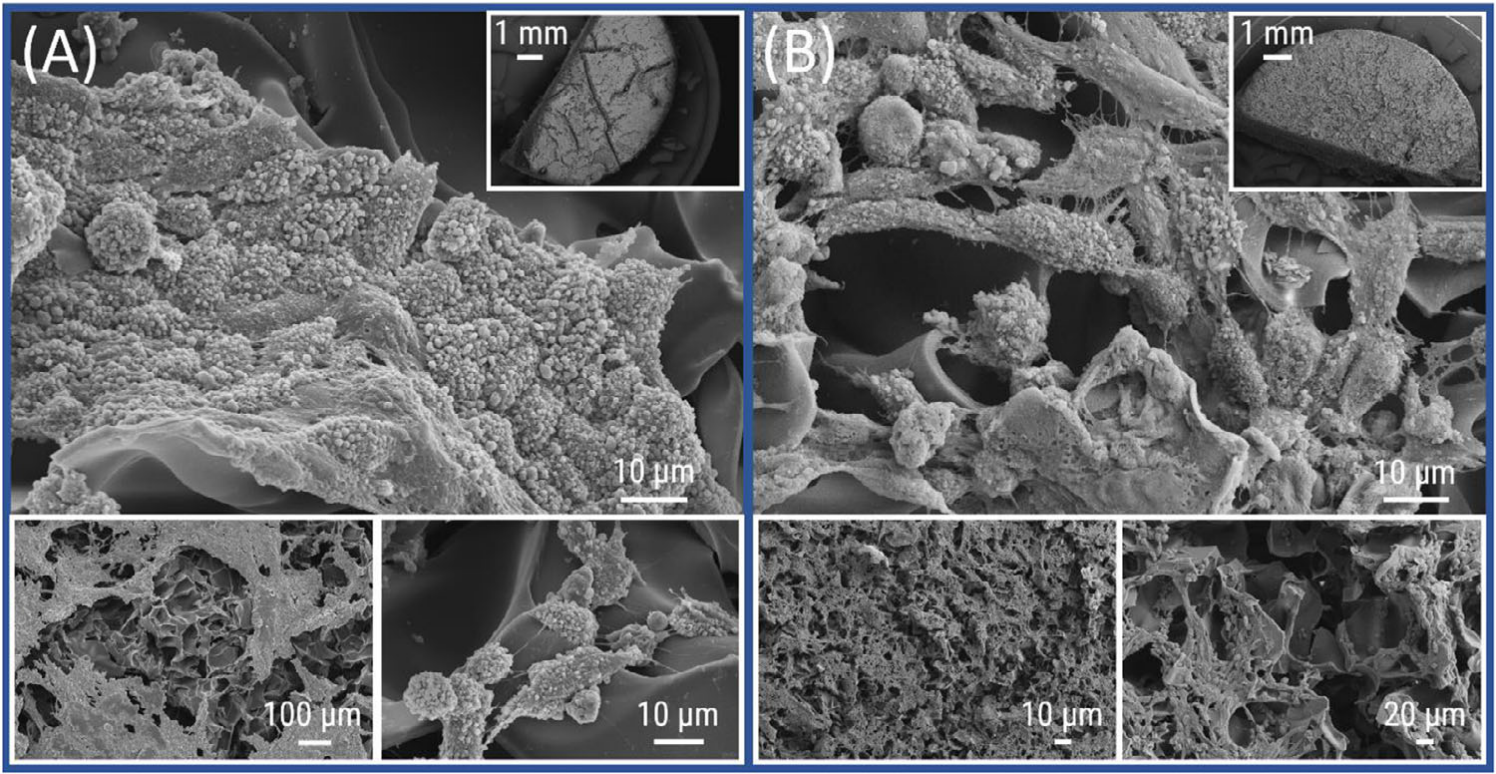
SEM micrographs of **CG(B‐Am‐PipA/RGD)** with different magnifications containing L929 cells which were fixed directly after cell culture on unwashed gels (A) or gels which were washed after the cell culture prior to the fixation (B) (A. top: 1000×; top right: 11×; bottom left: 100×; bottom rights: 1500×), (B. top: 1000×; top right: 12×; bottom left: 50×; bottom right: 250×).

Similar to **CG(B‐Am‐PipA)**, the gel **CG(B‐Am‐PipA/RGD)** showed no drastic differences between washed (Figure 9A) and unwashed gels (Figure 9B) indicating a strong cell‐cryogel interaction. The gels display adherent cells with predominantly flat morphologies but also to a minor degree with round shapes which is typical for cells entering the process of mitosis. Both cryogels variants **CG(B‐Am‐PipA)** and **CG(B‐Am‐PipA/RGD)** are exhibiting cytocompatibility because of the high cell density and increased degree of filopodia formation interacting with the gel surface as well as the apparent resemblance of observed cell morphology with conventional in vitro culture materials. No cells were detected at the bottom side of **CG(B‐Et‐PipA), CG(B‐Am‐PipA),** and **CG(B‐Am‐PipA/RGD)** gels (Figure S3). Interestingly, for **CG(B‐Am‐PipA)** and **CG(B‐Am‐PipA/RGD)**, cells could be detected on the outer vertical sides (Figures S4 and S5). This can most likely be attributed to cells reaching the side of the gel upon seeding and subsequent adhesion at these spots. Additionally, CLSM images of **CG(B‐Et‐PipA)RhoB, CG(B‐Am‐PipA)RhoB,** and **CG(B‐Am‐PipA/RGD)RhoB** were acquired after seven days of cocultivation with L929 cells, showing the presence of morphologically intact living cells in the porous structure of the cryogels (Figure S6).

## 3 Conclusion

In this study, we demonstrated the functionalization of POx‐based cryogels with an RGD‐peptide in a post‐fabrication functionalization process. The success of the RGD coupling was proven gravimetrically as well as by the use of CLSM, FT‐IR, and HR‐MAS measurements. The cryogels obtained in this manner demonstrated modulated physical properties. Rheological analyses revealed an increase in stiffness, while thermal gravimetric analyses (TGA) indicated enhanced thermal stability. On the other hand, the maximum swelling degree slightly decreased. A comparative cellular biological investigation was conducted using L929 cells, which were incubated with cryogel displaying ethyl groups (**CG(B‐ET‐PipA)**), aminopropyl groups (**CG(B‐Am‐PipA)**), and an RGD‐containing cell‐adhesive peptide sequence (**CG(B‐Am‐PipA/RGD)**). L929 cells were incubated with the samples **CG(B‐Et‐PipA)RhoB**, **CG(B‐Am‐PipA)RhoB**, and **CG(B‐Am‐PipA/RGD)RhoB** for 0.5, 1, 2, and 6 h to investigate their initial adhesion behavior using CLSM. Both **CG(B‐Am‐PipA)RhoB** and **CG(B‐Am‐PipA/RGD)RhoB** exhibited cell‐adhesive properties, with the RGD‐functionalized cryogel promoting faster adhesion and more pronounced cell spreading, reflected by larger cell areas. After 7 days of cultivation the use of scanning electron microscopy (SEM) imaging revealed that cells exhibited no notable adhesion on **CG(B‐Et‐PipA)**, in contrast to the observations for the other two types of cryogels **CG(B‐Am‐PipA)** and **CG(B‐Am‐PipA/RGD)**. Morphological analysis revealed that cells on the cryogel surface of **CG(B‐Am‐PipA)** and **CG(B‐Am‐PipA/RGD)** the presence of distinct spread cell bodies.

In future studies, the scope of this investigation could be expanded to other cell types, such as stem cells, to ascertain the impact of diverse surface functionalization on their differentiation. By systematically varying the amount of RGD used, a correlation between adhering cells and the amount of RGD could be established to identify the optimum conditions for cellular growth and differentiation.

## 4 Experimental Section/Methods

### 4.1 Materials

*N*,*N*,*N*’,*N*’‐Tetramethylethylenediamine (TMEDA, 99%, Sigma–Aldrich), potassium peroxodisulfate (K_2_S_2_O_8_, ≥ 98%, Fluka Analytical), acryloxyethyl thiocarbamoyl rhodamine B (RhoB, ≤ 100%, Polysciences), (FITC)‐GCWGRGDSP TFA salt (98%, Biomatik), GCWGRGDSP TFA salt (98%, Biomatik), 1‐ethyl‐3‐(3‐dimethylaminopropyl) carbodiimide hydrochloride (EDC‐HCl, 98%, TCI), *N*‐hydroxysuccinimide (NHS, 98%, Sigma–Aldrich), triethylamine (TEA, 99%, TCI) and PBS buffer (Capricorn Scientific) were used as purchased. All other chemicals and solvents were obtained from common commercial sources and were used without further purification unless stated otherwise.

### 4.2 Instruments

Microscopic evaluation of hydrated cryogels was performed using the confocal laser‐scanning microscope LSM880 Elyra PS.1 system (Zeiss, Oberkochen, Germany) with a C‐apochromate 40×/1.2 W Korr FCS M27 objective. Images were acquired and analyzed using the ZEN 3.8 software.

Scanning electron microscopy (SEM) micrographs of cell culture studies on cryogels were obtained using a Zeiss Sigma VP Field Emission Scanning Electron Microscope (Jena, Germany) with an SE‐2 detector and an accelerating voltage of 10 kV. For this purpose, dried cryogel slices were coated with a 9 nm platinum layer using a CCU‐010 HV Safematic sputter coater (Zizers, Switzerland) and fixed onto the plate holder pins with conductive carbon adhesive pads.

Proton nuclear magnetic resonance (^1^H) spectra were recorded on a 300 MHz spectrometer at room temperature equipped with an Avance I console and a dual proton probe 5 mm BBF‐1H/D z‐Grad probehead from Bruker. The residual ^1^H peak of D_2_O was used for chemical shift referencing. Chemical shifts are given in parts per million (ppm). High‐resolution magic‐angle‐spinning (HR‐MAS) NMR was recorded on a Bruker Avance III 500 MHz spectrometer with a 4 mm HR MAS probehead. The SamplePro automated sample loading system for 4 mm rotors was used.

Fourier‐transform infrared (FT‐IR) spectra were recorded from 400 to 4000 cm^−1^ using an IR‐Affinity‐1 CE system (Shimadzu, Kyoto, Japan) which was equipped with a quest ATR diamond extended range X‐single reflection‐ATR accessory with a diamond crystal.

The rheology measurements were performed on a MCR 301 rheometer from Anton Paar (Graz, Austria) using the convection oven device CTD 450. The samples were measured with a cone‐plate measuring setup with a cone angle of α = 1 (D‐CP15, Anton Paar (Graz, Austria)). The sample gap was set between 0.4 and 1.2 mm. The software RheoCompassTM V1.30.1064‐Release 64‐bit (Anton Paar, Graz, Austria) was applied for operating the rheometer as well as for the analysis. The data were exported as txt‐files and evaluated and processed with OriginPro 2022b (OriginLab Corporation, Northampton, MA, USA).

Thermogravimetric analysis (TGA) was carried out under nitrogen atmosphere using a Netzsch TG209 F1 Libra (Selb, Germany). For the measurements, a standard method was used as follows: Continuous heating from 25 to 600 °C with a heat rate of 20 K min^−1^; time: 29 min). If necessary, the data were smoothed after the measurements using the Netzsch Proteus Thermal Analysis Software Version 8.0.2.

Drying of cryogels was conducted by the use of an Alpha 2–4 LD plus freeze dryer from Martin Christ Gefriertrocknungsanlagen GmbH (Germany). For cell culture experiments, samples were dried by dehydration using a graded ethanol series (30%, 50%, 70%, 90%, and 100% ethanol in water) followed by critical point drying (Leica EM CPD300, Leica Microsystems, Wetzlar).

Ultrapure water was received from a Merck Millipore water purification system which was used for all cryogel preparations unless otherwise stated. For a precise and adjustable control of the reaction temperature for the cryopolymerization reactions, an ALPHA RA 8 cryostat from LAUDA was used.

### 4.3 General Procedure for Cryogel Preparation

The cryogel preparations were carried out in duplicates using 5 mL polypropylene syringes as reaction containers as described previously [16, 18]. In brief, an aqueous monomeric solution was prepared containing the cross‐linker and K_2_S_2_O_8_ followed by homogenization in an ultrasonic bath. The solution was then purged with argon at 0°C for 30 min before taking it up into the syringe. Subsequently, an aqueous *N*,*N*,*N*’,*N*’‐tetramethylethylenediamine solution (80 µL of a 1.114 m solution, 0.089 mmol) was added through the bottom end followed by bottom‐capping using a syringe stopper and vortexing for 10 s. For cryopolymerization, the capped syringe was placed in a cryostat cooling bath (−12 °C) overnight using a perforated polystyrene grid. Upon removal from the cryostat bath and thawing at room temperature for 1 h, the resulting cryogel was immersed in water, with subsequent solvent changes for one day. Cut cryogel slices were freeze‐dried for at least two days. Three or four slices were ground to a fine powder for HR‐MAS NMR and thermogravimetric analysis. The exact experimental details are summarized in Table S1.

### 4.4 Cryogel Functionalization with RGD

The functionalization of cryogels with GCWGRGDSP (**RGD**) was accomplished through the use of seven pre‐dried **CG(B‐Am‐PipA)** gels. A solution was prepared by dissolving 64 mg of **RGD**, 107 mg of EDC‐HCl, and 107 mg of NHS in 2.7 mL of deionized water, followed by stirring. TEA (107 µL) was then added, and 380 µL of the reaction mixture was transferred to each cryogel. The gels were left to stand overnight. Subsequently, the cryogels were washed thrice with water, thrice with a triethylamine solution, and once more with water. Subsequently the gels were dehydrated using an ethanol dehydration series (30%, 50%, 70%, 90%, 100%). The gels were then dried using a critical point dryer.

In case of the rhodamine labelled gels, 10 mg of EDC‐HCl and 10 mg of NHS were dissolved in 250 µL of water, before 8 mg of the FITC containing peptide FITC‐GCWGRGDSP (**FITC‐RGD**) was added. The resulting mixture was stirred, and TEA (10 µL) was added. The reaction mixture was subsequently added to dry **CG(B‐Am‐PipA)RhoB** and left to stand in the dark for two days. Subsequently, the solution underwent a series of washing steps, including water (three times), ethanol (once), TEA solution (once), and water again (once). The success of the functionalization with **FITC‐RGD** was proven *via* CLSM, employing previously prepared rhodamine‐labeled gels [18].

### 4.5 Confocal Laser‐Scanning Microscopy of RGD Labelled Cryogels

For excitation of FITC and RhoB a 488 and a 561 nm laser were used, respectively. Fluorescence emission was detected in single track mode with a 32 channel GaAsP detector using a filter−free adjustable prism optic to detect the desired wavelength range of λ_em_ FITC = 493 to 562 nm and λ_em_ RhoB = 566 to 703 nm. Main beam path splitter at 488 and 561 nm was utilized to exclude excitation light from detection. Colocalization was visualized in overlay images of the three channels and additionally quantified pixelwise using the colocalization tool of the Zen‐software. Based on the individual fluorescence properties of each pixel and a threshold to exclude pixels with none or very low fluorescence intensities, three regions were generated (1 = only FITC signal; 2 = only RhoB signal; 3 = both FITC and RhoB signal) and could be used for colour coded visualization and quantification of colocalized signals as well as determination of the colocalization coefficient.

### 4.6 Rheological Measurements

Rheological measurements were performed according to a literature procedure [27]. The storage modulus G’ was determined by frequency sweeps performed in the range of 0.03 to 1.0 Hz at 25 °C with an oscillating shear deformation of 0.005%. The two cryogel samples **CG(B‐Am‐PipA)** and **CG(B‐Am‐PipA/RGD)** (*n* = 3) were swollen in distilled water and hereafter transferred into the rheometer. To prevent the cryogels from dehydration during the measurement the samples were surrounded with distilled water.

### 4.7 Swelling Behavior

The swelling behavior of cryogels was studied as described in the literature at room temperature by sampling a circular piece of the dry cryogel (*n* = 3) in a vial containing deionized water [28]. At periodic time points (20, 40, 60, 300, 1800 s), the increase in the mass of the cryogel sample was documented after the removal of the excess water that had adhered to the surface.

The water uptake was calculated as the cryogel swelling ratio according to the following Equation (1):

(1) $$\mathrm{CG}\;\mathrm{swelling}\;\mathrm{ratio}\;=\frac{m{\left(\mathrm{CG}\right)}_{\mathrm{wet}}-m{\left(\mathrm{CG}\right)}_{\mathrm{dry}}}{m{\left(\mathrm{CG}\right)}_{\mathrm{dry}}}\;$$

where m(CG)_wet_ and m(CG)_dry_ correspond to the masses of the cryogels in their swollen and dried states, respectively.

### 4.8 Cell Culture and Cultivation with Cryogels

Prior to cultivation experiments, cryogels were sterilized with 70% EtOH for 24 h and subsequently washed three times with PBS. L929 fibroblast cells were routinely cultured as follows: Dulbecco's modified eagle's medium (DMEM) supplemented with 10% fetal calf serum (FCS), 1 g/L glucose, 100 U/mL penicillin, and 100 µg/mL streptomycin (D10F+, all components from Biochrom, Berlin, Germany) at 37°C in a humidified atmosphere with 5% (v/v) CO_2_. After cell detachment using trypsin treatment, cells for the CLSM study were suspended in serumfree DMEM (2 × 10^6^ cells/mL) containing Hoechst 33258 (1 µg/mL) and CelltrackerDeepred (CTDR) (10 µm) and were incubated at 37 °C for 30 min to label nuclei and cytoplasm, respectively [10]. After centrifugation cells were resuspended in D10F+ to yield a cell concentration of 1 × 10^5^ cells/mL. Cells prepared for SEM analysis were left unstained and were directly resuspended in D10F+ to yield a cell concentration of 1 × 10^5^ cells/mL. The cryogel slices were equilibrated in D10F+ for 1 h and subsequently transferred into 5 mL polypropylene syringes carrying a bottom cap. The gels were tightly fitting to the sidewalls of the sterile syringes mitigating an unhindered cell sedimentation aside the cryogels. 5 mL of the cell suspension were applied onto each cryogel (5 × 10^5^ cells per cryogel slice). After adding the cell suspension, syringes were transferred to a 50 mL falcon tube which was loosely capped to ensure gas permeation. The following incubation was carried out at 37 °C in a humidified atmosphere with 5% (v/v) CO_2_. Next, the supernatant containing cell culture media was carefully aspirated. Cryogels were taken out of the syringe and either directly subjected to CLSM analysis or further treated for SEM studies. CLSM samples were directly transferred topside down into 24‐well ibidi microscope plates (Sarstedt, Numbrecht, Germany) followed by the addition of PBS buffer to avoid dehydration of the slices. For SEM, samples were cut vertically into two pieces, and one piece was directly subjected to further sample preparation for microscopic evaluation. The second piece was dipped three times into PBS to remove unbound or loosely attached cells. Samples were fixed for 1 h using a solution of 2% glutaraldehyde in PBS and washed three times with PBS. Next a dehydration was performed using an ethanol series (30%, 50%, 70%, 90%, 100%). After critical point drying, samples were cut vertically in half along its central axis, and the top and bottom sections were examined separately by SEM upon coating.

### 4.9 Confocal Laser‐Scanning Microscopy of Cryogels Cultured with Cells

For excitation of Hoechst 33258, RhoB, and CTDR a 405, a 514, and a 633 nm laser were used, respectively. Fluorescence emission was detected in single track mode with a 32 channel GaAsP detector using a filter−free adjustable prism optic to detect the desired wavelength range (λ_em,Hoechst 33258_ = 410 to 546 nm, λ_em, RhoB_ = 535 to 668 nm, λ_em,CTDR_ = 661 to 749 nm). Main beam path splitter at 405, 514, and 633 nm was utilized to exclude excitation light from detection. Colocalization was visualized in overlay images of the three channels.

## Funding

This work was funded by the German Research Foundation (DFG, project number: SCHU 1229/25‐2 and LI 916/19‐2) and under Germany's Excellence Strategy (EXC 2051 project number: 390713860) as well as the Federal State of Thuringia (Germany) and the European Union within the framework of the European Regional Development Fund (ERDF) (2016 IZN 0009). The SEM facilities of the Jena Center for Soft Matter (JCSM) were established with a grant from the DFG. Acquisition of the 500 MHz NMR spectrometer and the CLSM were supported by the grants ‘Forschungsgroßgeräte (FuGG)’, INST 275/331‐1 FUGG and INST 275/315‐1 FUGG of the German Research Foundation (DFG).

## Conflicts of Interest

The authors declare no conflicts of interest.

## Supporting information




**Supporting File**: mabi70124‐sup‐0001‐SuppMat.docx.

## Data Availability

The data which are supporting the study's findings are available from the corresponding author upon reasonable request.
