# Supplementary material for: RGD Modification of Poly(2‐oxazoline) Cryogels: Investigation of Material Properties and Cellular Adhesion
Source: Macromol Biosci. 2026 Jan 14;26(1):e00421. doi: 10.1002/mabi.202500421 (PMC12800887; doi:10.1002/mabi.202500421)
Supplement: Supplementary file 1 — Supporting File: mabi70124‐sup‐0001‐SuppMat.docx. [file MABI-26-e00421-s001.docx]

Supporting Information

RGD modification of poly(2-oxazoline) cryogels: Investigation of material properties and cellular adhesion

Tim Hoffmann, David Pretzel, Steffi Stumpf, Florian Behrendt, Michael Klein, Leon Lange, Lena-Marie Kaspar, Klaus Liefeith, Michael Gottschaldt, Ulrich S. Schubert*

Tim Hoffmann, David Pretzel, Steffi Stumpf, Florian Behrendt, Michael Klein, Leon Lange, Lena-Marie Kaspar, Michael Gottschaldt, Ulrich S. Schubert

Laboratory of Organic Chemistry and Macromolecular Chemistry (IOMC), Friedrich Schiller University Jena, Humboldtstraße 10, 07743 Jena, Germany

Jena Center for Soft Matter (JCSM), Friedrich Schiller University Jena, Philosophenweg 7, 07743 Jena, Germany

Email: [ulrich.schubert@uni-jena.de](mailto:ulrich.schubert@uni-jena.de)

Florian Behrendt, Ulrich S. Schubert

Cluster of Excellence Balance of the Microverse, Friedrich Schiller University Jena, Jena, Germany

Klaus Liefeith

Institute for Bioprocessing and Analytical Measurement Techniques e.V., Rosenhof, 37308 Heilbad Heiligenstadt, Germany

[1 Cryogel preparation 4](#_Toc209613105)

[2 Cell culture experiments 6](#_Toc209613106)

[2.1 Scanning electron microscopy (SEM) 6](#_Toc209613107)

[2.2 Confocal laser-scanning microscopy (CLSM) 9](#_Toc209613108)

# Cryogel preparation

The cryogel preparations were carried out in duplicates using 5 mL polypropylene syringes as reaction containers as described previously.^[1-2]^ In brief, an aqueous monomeric solution was prepared containing the cross-linker and K_2_S_2_O_8_ followed by homogenization in an ultrasonic bath. The solution was then purged with argon at 0 °C for 30 min before taking it up into the syringe. Subsequently, an aqueous *N*,*N*,*N*’,*N*’-tetramethylethylenediamine (TMEDA) solution (80 µL of a 1.114 M solution, 0.089 mmol) was added through the bottom end followed by bottom-capping using a syringe stopper and vortexing for 10 s. For cryopolymerization, the capped syringe was placed in a cryostat cooling bath (–12 °C) overnight using a perforated polystyrene grid. Upon removal from the cryostat bath and thawing at room temperature for 1 h, the resulting cryogel was immersed in water, with subsequent solvent changes for one day. Cut cryogel slices were freeze-dried for at least two days. Three or four slices were ground to a fine powder for HR-MAS NMR and thermogravimetric analysis. The exact experimental details are summarized in **Table S1**.

**Table S1**. Overview about the conditions and parameters for the preparation of cryogels using **B-Et-PipA** and **B-Am-PipA** as cross-linker. The amounts of cross-linker and K_2_S_2_O_8_ (11.1 mM, 0.089 mmol) are given per 8 mL which corresponds to one duplicate of 5 mL syringes each. Individual volume in each syringe was 3.98 mL after addition of TMEDA. t_R_ = reaction time. [M] = concentration of monomer in the syringe. [I] = initiator concentration in the syringe.

| CL | Cryogel name | CL amount | t_R_  [h] | [M]  [mol L^-1^] | [I]  [mol L^-1^] |
| --- | --- | --- | --- | --- | --- |
| B-Am-PipA | **CG(B-Am-PipA)** | 405 mg  0.225 mmol | 20 | 0.028 | 0.011 |
| B-Et-PipA | **CG(B-Et-PipA)** | 309 mg  0.225 mmol | 20.5 | 0.028 | 0.011 |

The functionalization of cryogels with GCWGRGDSP (**RGD**) was accomplished through the use of seven pre-dried **CG(B-Am-PipA)** gels. A solution was prepared by dissolving 64 mg of **RGD**, 107 mg of 1-ethyl-3-(3-dimethylaminopropyl) carbodiimide hydrochloride, and 107 mg of *N*-hydroxysuccinimide in 2.7 mL of deionized water, followed by stirring. Triethylamine (107 µL) was then added, and 380 µL of the reaction mixture was transferred to each cryogel. The gels were left to stand overnight. Subsequently, the cryogels were washed thrice with water, thrice with a triethylamine solution, and once more with water. Subsequently the gels were dehydrated using an ethanol dehydration series (30%, 50%, 70%, 90%, 100%). The gels were then dried using a critical point dryer.

**Table S2.** Overview about the experimentally determined masses for the coupling reaction and the resulting calculated **RGD** content.

|  | CG(B-Am-PipA) mass [mg] | CG(B-Am-PipA/RGD) [mg] | RGD peptide per polymerchain  (cross-linker) | RGD mol% |
| --- | --- | --- | --- | --- |
| 1 | 15.51 | 17.10 | 0.83 | 45 |
| 2 | 16.19 | 17.58 | 0.80 | 44 |
| 3 | 13.73 | 15.21 | 0.85 | 46 |
| 4 | 15.71 | 17.38 | 0.84 | 45 |
| 5 | 15.60 | 17.15 | 0.83 | 45 |
| 6 | 18.44 | 20.16 | 0.81 | 45 |
| 7 | 18.57 | 20.15 | 0.80 | 44 |


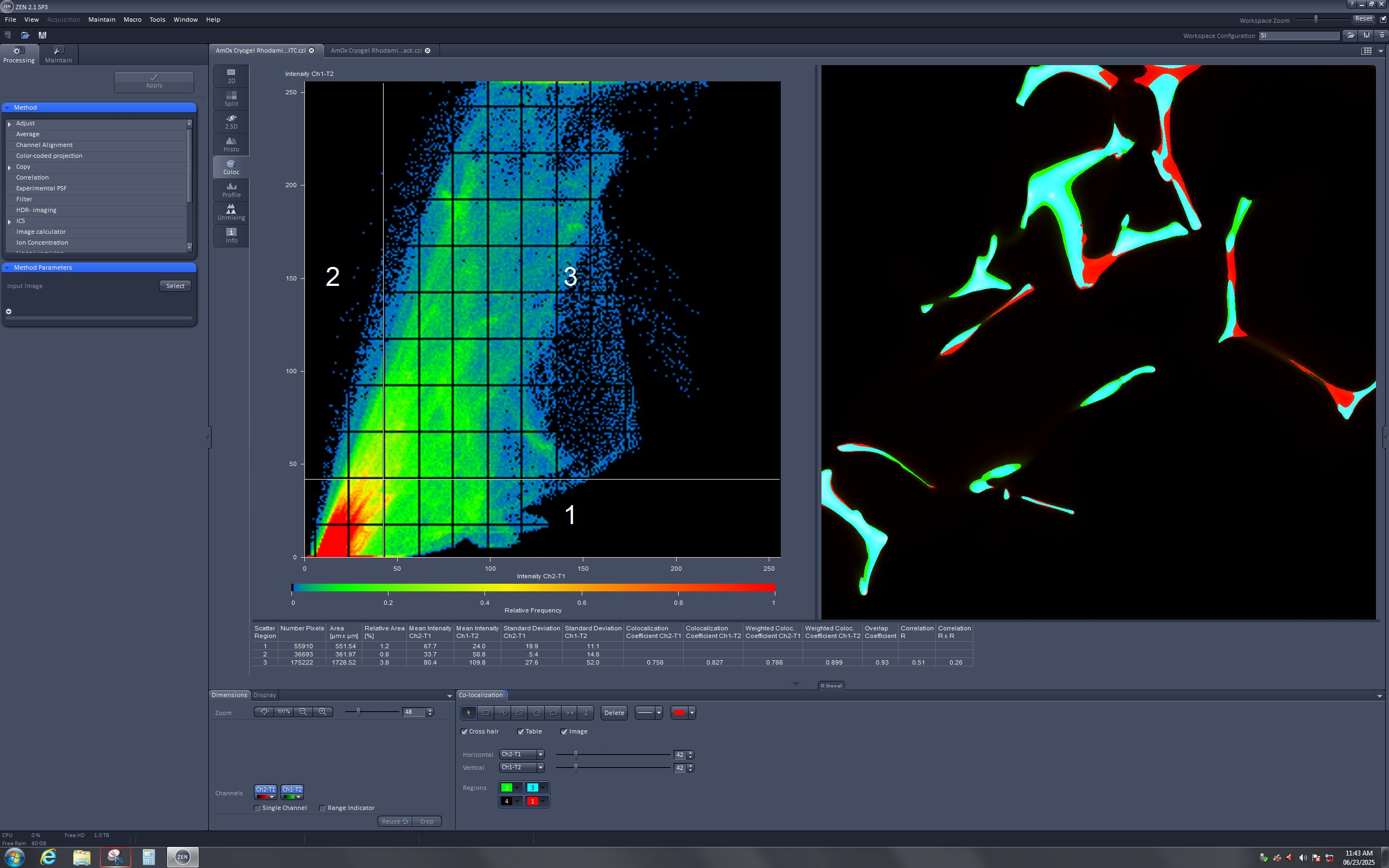


**Figure S1:** Scatter diagram and corresponding color-coded image of colocalization of **CG(B-Am-PipA/RGD).** Individual pixelwise fluorescein (FITC) labelled peptide ((FITC)-GCWGRGDSP) and acryloxyethyl thiocarbamoyl rhodamine B (RhoB) (cryogel) fluorescence intensities were used for identification of non-colocalized (region 1 = green - only FITC signal; region 2 = red - only RhoB signal) and colocalized signals (region 3 = cyan – both FITC and RhoB signal).

# 2 Cell culture experiments

Prior to cultivation experiments, cryogels were sterilized with 70% EtOH for 24 h and subsequently washed three times with PBS. L929 fibroblast cells were routinely cultured as follows: Dulbecco’s modified eagle’s medium (DMEM) supplemented with 10% fetal calf serum (FCS), 1 g/L glucose, 100 U/mL penicillin and 100 µg/mL streptomycin (D10F+, all components from Biochrom, Berlin, Germany) at 37 °C in a humidified atmosphere with 5% (v/v) CO_2_. After cell detachment using trypsin treatment, cells for the CLSM study were suspended in serumfree DMEM (2×10^6^ cells/mL) containing Hoechst 33258 (1 µg/mL) and CelltrackerDeepred (CTDR) (10 µM) and were incubated at 37 °C for 30 min to label nuclei and cytoplasm, respectively.^[3]^ After centrifugation cells were resuspended in D10F+ to yield a cell concentration of 1×10^5^ cells/mL. Cells prepared for SEM analysis were left unstained and were directly resuspended in D10F+ to yield a cell concentration of 1×10^5^ cells/mL. The cryogel slices were equilibrated in D10F+ for 1 h and subsequently transferred into 5 mL polypropylene syringes carrying a bottom cap. The gels were tightly fitting to the sidewalls of the sterile syringes mitigating an unhindered cell sedimentation aside the cryogels. 5 mL of the cell suspension were applied onto each cryogel (5×10^5^ cells per cryogel slice). After adding the cell suspension, syringes were transferred to a 50 mL falcon tube which was loosely capped to ensure gas permeation. The following incubation was carried out at 37 °C in a humidified atmosphere with 5% (v/v) CO_2_. Next, the supernatant containing cell culture media was carefully aspirated. Cryogels were taken out of the syringe and either directly subjected to CLSM analysis or further treated for SEM studies. CLSM samples were directly transferred topside down into 24-well ibidi microscope plates (Sarstedt®, Numbrecht, Germany) followed by the addition of PBS buffer to avoid dehydration of the slices. For SEM, samples were cut vertically into two pieces and one piece was directly subjected to further sample preparation for microscopic evaluation. The second piece was dipped three times into PBS to remove unbound or loosely attached cells. Samples were fixed for 1 h using a solution of 2% glutaraldehyde in PBS and washed three times with PBS. Next a dehydration was performed using an ethanol series (30%, 50%, 70%, 90%, 100%). After critical point drying, samples were cut vertically in the half along its central axis and the top and bottom sections were examined separately by SEM upon coating.

## 2.1 Scanning electron microscopy (SEM)


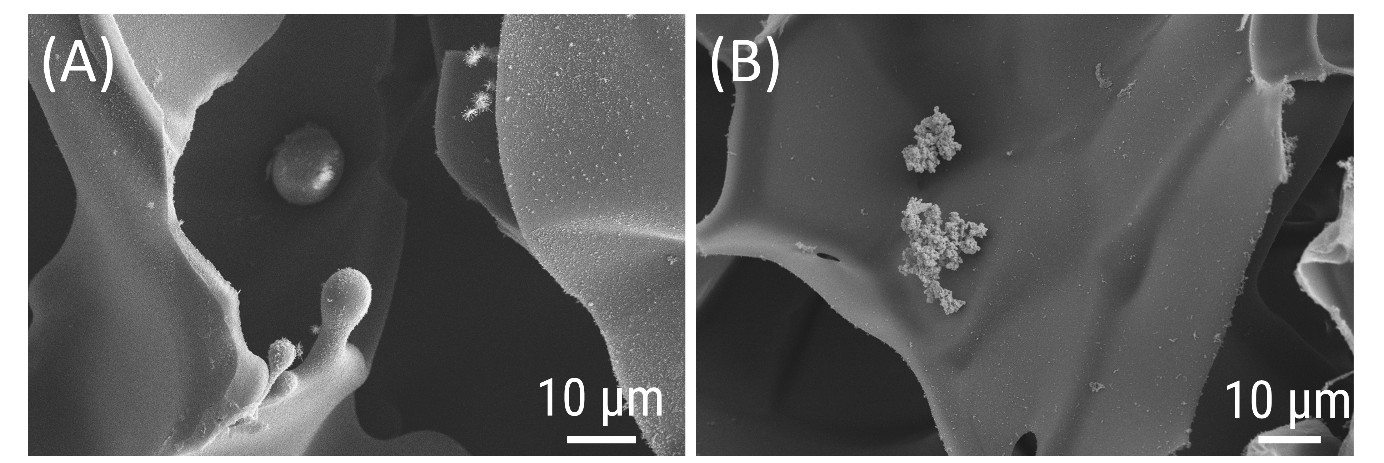


**Figure S2**: SEM micrographs of **CG(B-Et-PipA)** with different magnifications after application of cell medium without cells (A: 1190x, B: 1060x).


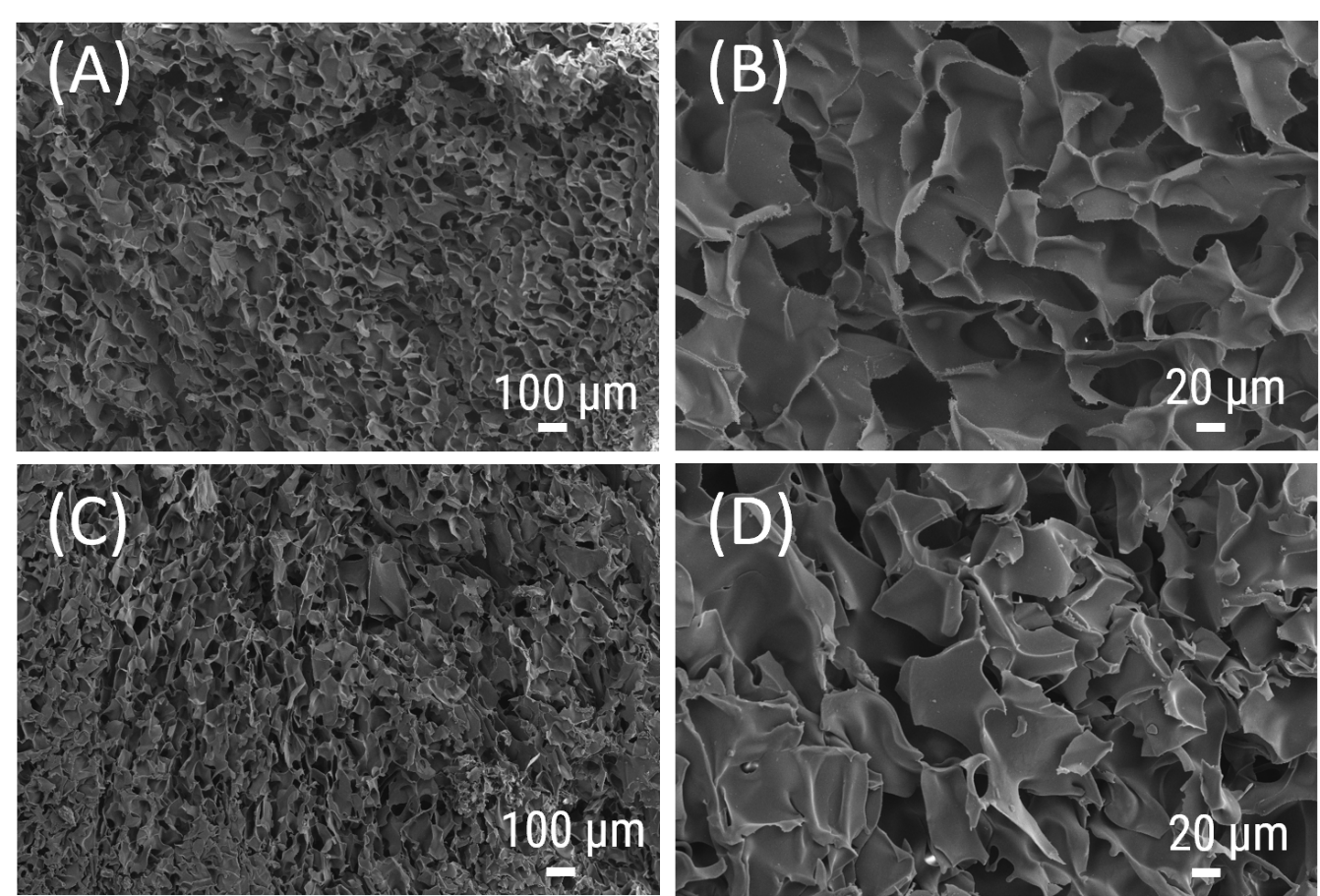


**Figure S3**: SEM micrographs of **CG(B-Et-PipA)** (bottom side of the slice), with different magnifications containing L929 cells which were fixed directly after cell culture on unwashed gels (A, B) or gels which were washed after the cell culture prior to the fixation (C, D) (A: 50x; B: 250x; C: 50x; D: 250x).


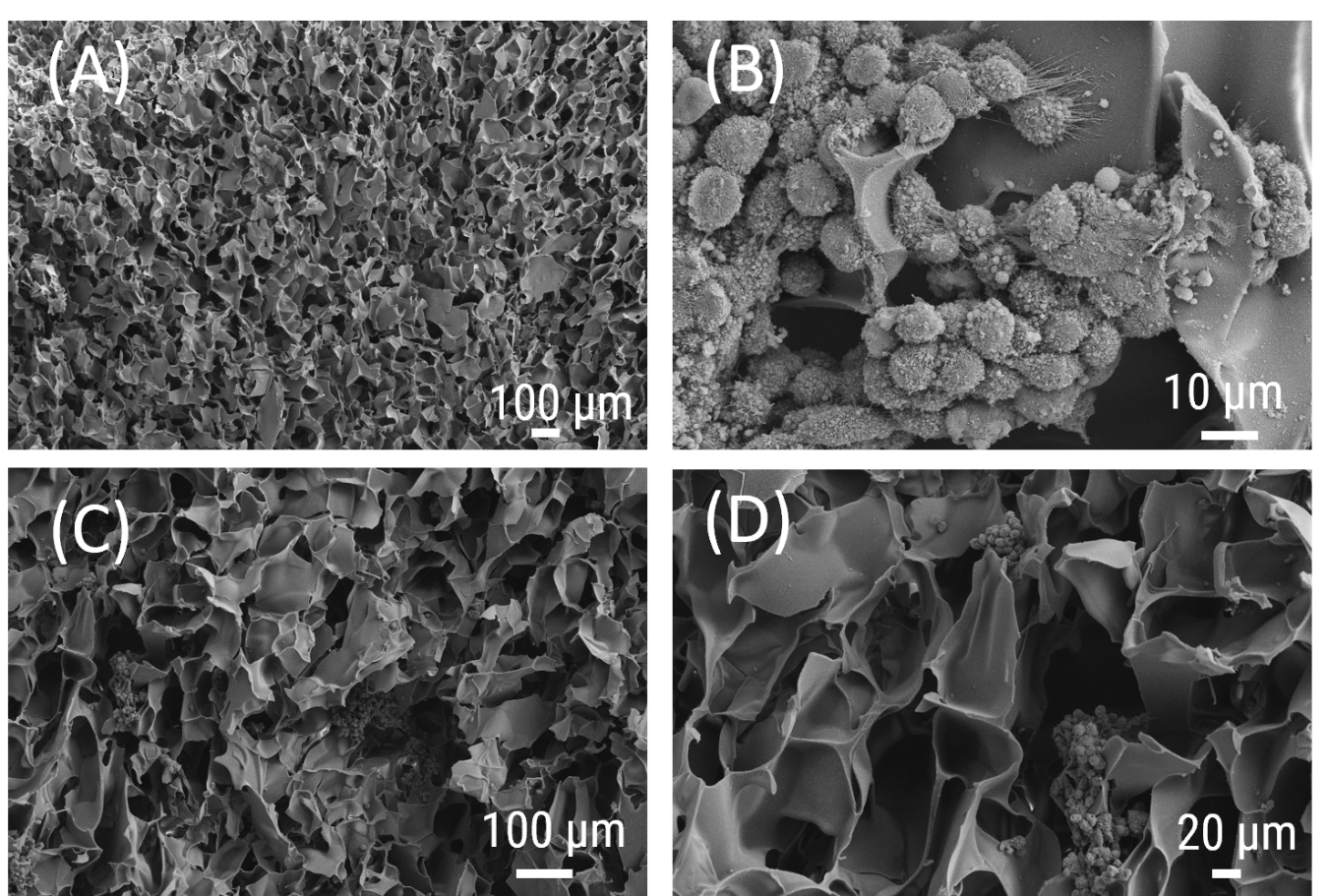


**Figure S4:** SEM micrographs of **CG(B-Am-PipA)** (bottom side of the slice), with different magnifications containing L929 cells which were fixed directly after cell culture on unwashed gels (A, B) or gels which were washed after the cell culture prior to the fixation (C, D) (A: 50x; B: 1000x; C: 100x; D: 250x).


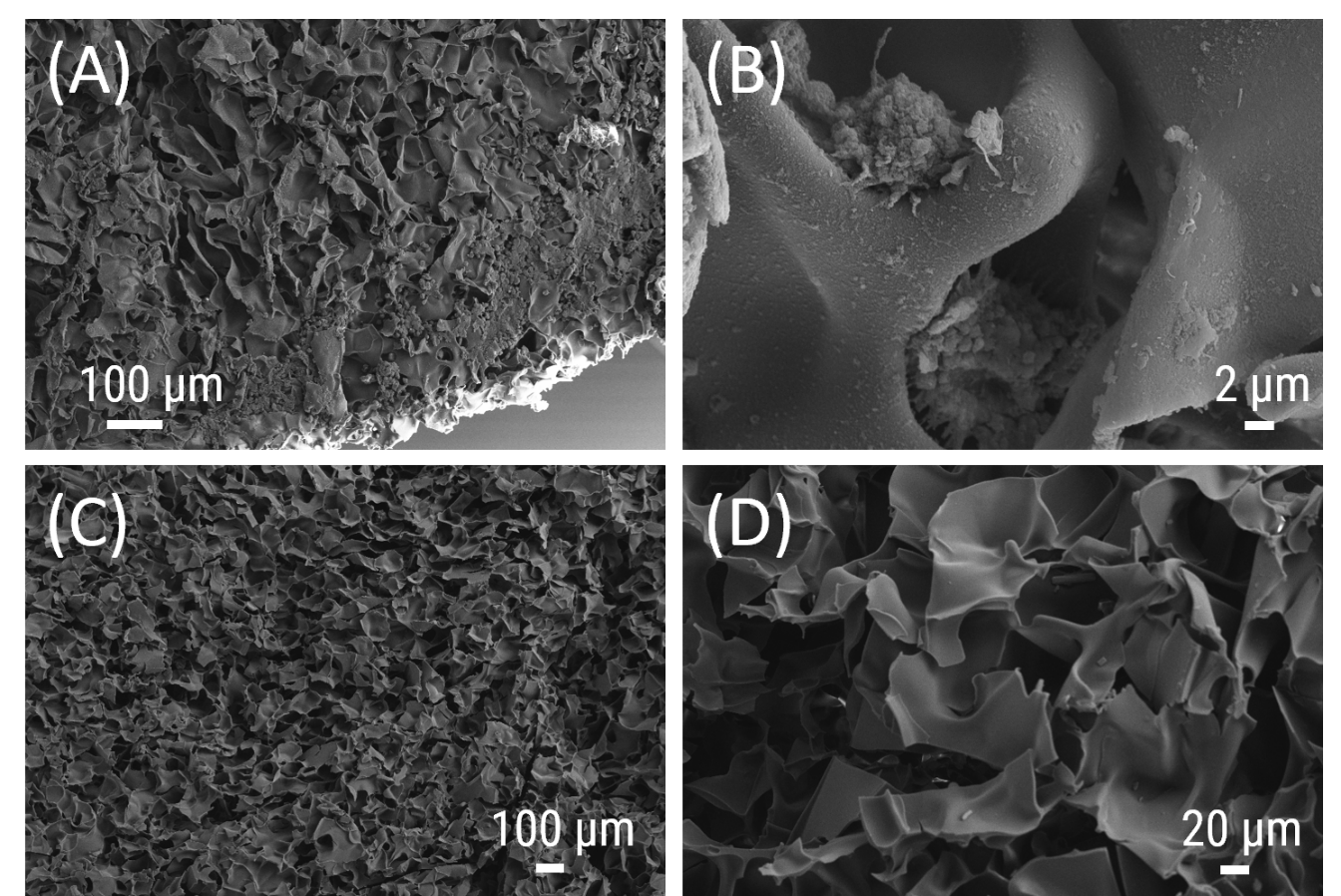


**Figure S5**: SEM micrographs of **CG(B-Et-PipA)** (bottom side of the slice), with different magnifications containing L929 cells which were fixed directly after cell culture on unwashed gels (A, B) or gels which were washed after the cell culture prior to the fixation (C, D) (A: 100x; B: 2500x; C: 50x; D: 250x).

## 2.2 Confocal laser-scanning microscopy (CLSM)

Microscopic evaluation of hydrated cryogels was performed using the confocal laser-scanning microscope LSM880 Elyra PS.1 system (Zeiss, Oberkochen, Germany) with a C-apochromate 40×/1.2 W Korr FCS M27 objective. Images were acquired and analyzed using the ZEN 3.8 software.


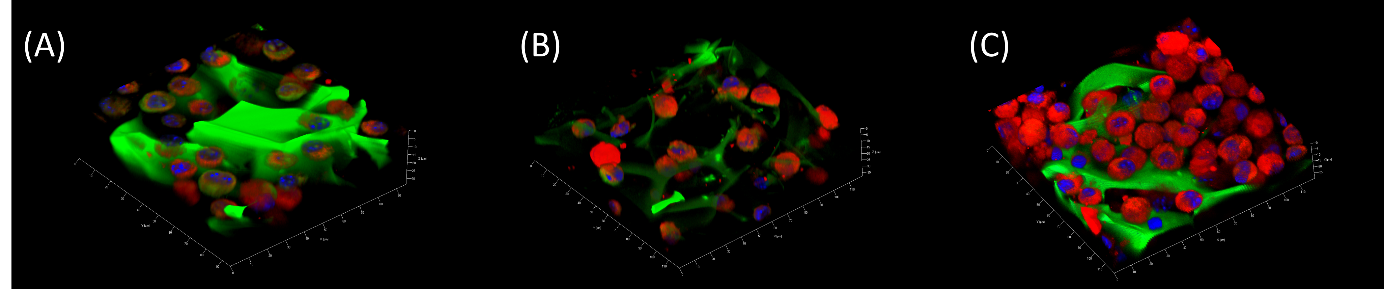
**Figure S6:** CLSM images of cryogels (A: **CG(B-Et-PipA)**, B: **CG(B-Am-PipA)**, C: **CG(B-Am-PipA/RGD)** after 7 days of coculture with L929 fibroblast cells. Red signal shows the cell plasma and blue signal displays cell nuclei.

References

[1] Engel, N., Hoffmann, T., Behrendt, F., Liebing, P., Weber, C., Gottschaldt, M., Schubert, U. S., Cryogels based on poly(2-oxazoline)s through development of bi- and trifunctional cross-linkers incorporating end groups with adjustable stability, 2024, Macromolecules, 57, 2915-2927, <https://doi.org/10.1021/acs.macromol.3c02030>

[2] Hoffmann, T., Behrendt, F., Klein, M., Lange, L., Engel, N., Pretzel, D., Czich, S., Liefeith, K., Gottschaldt, M., Schubert, U. S., Adjustment of physical properties and pH-responsive behavior of cryogels based on two- and three-armed poly(2-oxazoline) cross-linkers with functional side chains, 2025, Eur. Polym. J., 114031, <https://www.sciencedirect.com/science/article/pii/S0014305725003192>

­­
